# Application of amide hydrogen/deuterium exchange mass spectrometry for epitope mapping in human cystatin C

**DOI:** 10.1007/s00726-016-2316-y

**Published:** 2016-08-29

**Authors:** Martyna Prądzińska, Izabela Behrendt, Juan Astorga-Wells, Aleksandr Manoilov, Roman A. Zubarev, Aleksandra S. Kołodziejczyk, Sylwia Rodziewicz-Motowidło, Paulina Czaplewska

**Affiliations:** 1 Faculty of Chemistry, Department of Biomedical Chemistry, University of Gdańsk, Wita Stwosza 63, 80-952, Gdańsk, Poland; 2 Division of Physiological Chemistry I, Department of Medical Biochemistry and Biophysics, Karolinska Institutet, Scheeles väg 2, S-171 77 Stockholm, Sweden; 3 Biomotif AB, 18212 Stockholm, Sweden; 4 Intercollegiate Faculty of Biotechnology, University of Gdańsk-Medical University of Gdańsk, Kładki 24, 80-822 Gdańsk, Poland

**Keywords:** Human cystatin C, HDX exchange, Mass spectrometry, Epitope identification

## Abstract

Human cystatin C (hCC) is a small cysteine protease inhibitor whose oligomerization by propagated domain swapping is linked to certain neurological disorders. One of the ways to prevent hCC dimerization and fibrillogenesis is to enable its interaction with a proper antibody. Herein, the sites of interaction of hCC with dimer-preventing mouse monoclonal anti-hCC antibodies Cyst28 are studied and compared with the binding sites found for mAb Cyst10 that has almost no effect on hCC dimerization. In addition, hCC epitopes in complexes with native polyclonal antibodies extracted from human serum were studied. The results obtained with hydrogen–deuterium exchange mass spectrometry (HDX MS) were compared with the previous findings made using the excision/extraction MS approach. The main results from the two complementary MS-based approaches are found to be in agreement with each other, with some differences being attributed to the specificity of each method. The findings of the current studies may be important for future design of hCC dimerization inhibitors.

**Electronic supplementary material:**

The online version of this article (doi:10.1007/s00726-016-2316-y) contains supplementary material, which is available to authorized users.

## Introduction

**Fig. 1:**
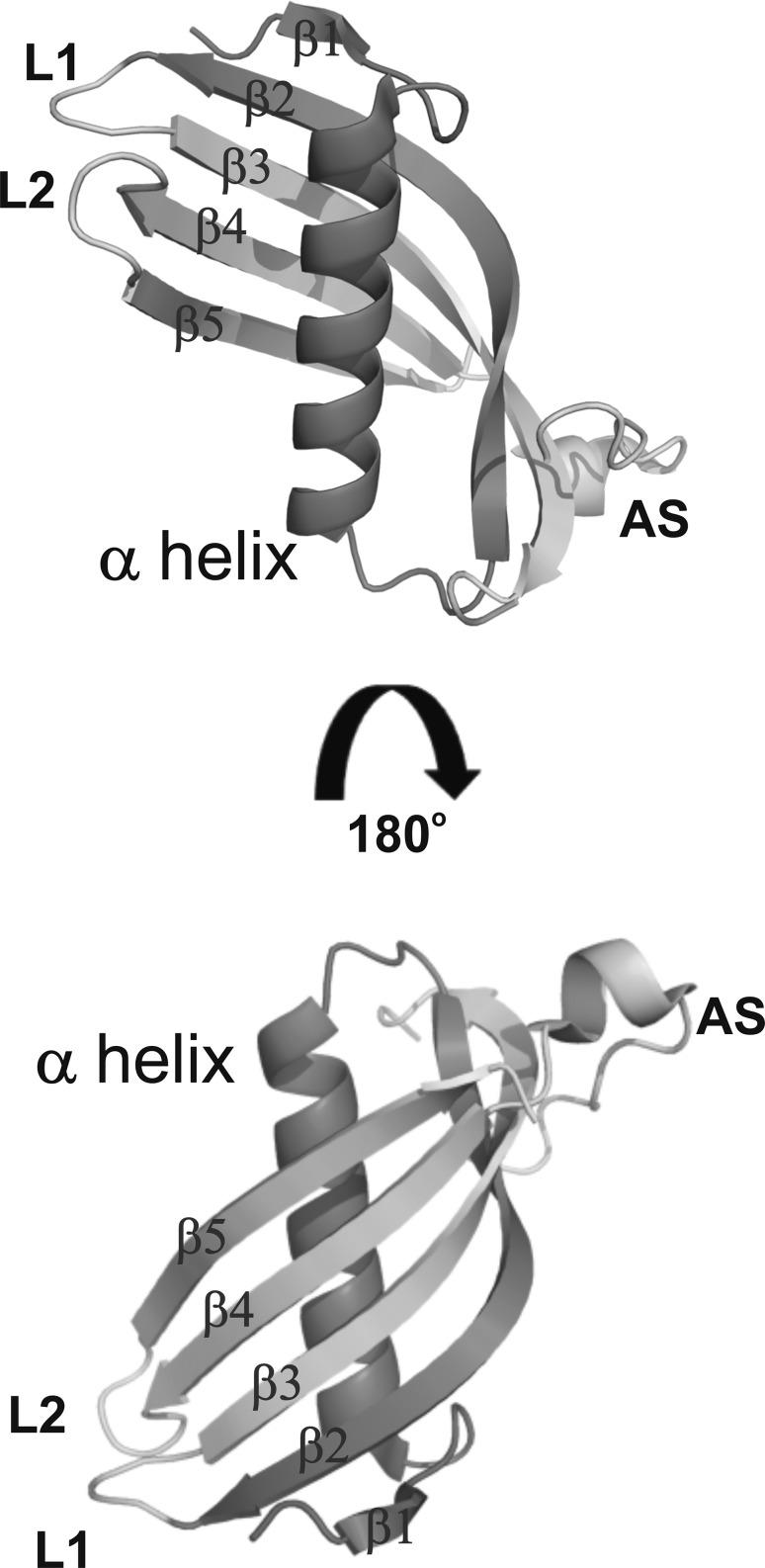
Monomeric structure of human cystatin C (PDB: 3GAX). L1, loop 1; L2, loop 2; β1–5, beta strands; AS, appendix structure. Structures involved in 3D domain swapping process are matched in *red*

Human cystatin C (hCC) belongs to a large group of cysteine protease inhibitors. This small, non-glycosylated protein consisting of 120 amino acids is present in all body fluids. However, it occurs in a highest concentration in cerebrospinal fluid, seminal plasma and milk (Mussap and Plebani 2004). The structure of the protein, stabilized by two disulfide bridges between residues 73–83 and 97–117, is well defined and consists of a five-stranded anti-parallel β-sheet (β1–β5) surrounding an α-helix, two hairpin loops (L1 and L2) and the so-called “appending structure” (AS). The latter is unrelated to the compact core of the molecule and positioned on the opposite end of the β-sheet relative to the N-terminus and loops L1 and L2 (Bode et al. 1988; Martin et al. 1995; Szymańska et al. 2009) (Fig. 1). Even though cystatin C is quite stable in the monomeric state, the crystal structure of hCC was difficult to obtain as it forms covalently bound dimers by exchange of two subdomains of the monomeric protein. This process, called “domain swapping,” has also been suggested to be involved in generation of amyloid fibrils making cystatin C a member of the amyloidogenic protein family.

In the Icelandic population, naturally occurring hereditary point mutation in the CST3 gene leads to the emergence of the pathological L68Q variant of hCC (Palsdottir et al. 1989). The substitution of hydrophobic leucine for hydrophilic glutamine in position 68 causes spontaneous protein dimerization and determines highly amyloidogenic properties of the variant (Wahlbom et al. 2007). Cystatin C dimers, especially of the L68Q type, can easily associate in oligomeric structures and then form higher amyloidogenic aggregates (Abrahamson and Grubb 1994). These aggregates are the main reason for brain hemorrhages at young age and high death rate among patients suffering from specific angiopathy called hereditary cystatin C amyloid angiopathy (HCCAA) (Jensson et al. 1987; Grubb 2000; Calero et al. 2001; Olafsson and Grubb 2000). Until now, there is no effective cure for this neurodegenerative disorder; however, it was reported that exogenous agents like monoclonal antibody against cystatin C are able to suppress formation of cystatin C dimers. Therefore, anti-cystatin C antibodies can be the hope for patients suffering from hereditary cystatin C amyloid angiopathy.

Currently, immunotherapies are among main trends in neurodegenerative diseases treatment (Valera and Masliah 2013). It seems that the pharmacological strategies used, e.g., in the fight against Alzheimer’s Disease (AD) only slightly slow down the process of the disease development, while immunotherapies show the potential to suppress the disease progression in many AD patients (Davtyan et al. 2014).

The present work describes our quest to find an effective inhibitor of hCC fibrillogenesis by studying natural complexes of cystatin C with different antibodies. Identification of all antigenic determinants on hCC surface may be a starting point to immunotherapy in HCCAA.

In studies carried out by Östner and collaborators, three out of the twelve tested monoclonal antibodies against hCC were identified as significantly suppressing dimer formation (HCC3, Cyst16, and Cyst28), whereas the Cyst10 clone was found to have almost no influence on the dimerization process (Östner et al. 2011). The hCC binding sites (epitopes) for two antibodies with opposite antiaggregational potential, Cyst10 and Cyst28, have already been identified using MS-assisted limited proteolysis (epitope excision or extraction procedures) (Behrendt et al. 2016). The same method has been used in our previous work on epitope identification for the monoclonal anti-cystatin antibody Cyst13 (Hager-Braun and Tomer 2005), which represents moderate inhibitory properties (Östner et al. 2011). The epitope excision mass spectrometry approach is based on the formation of a complex between an immobilized antibody and an antigen, followed by its digestion by proteolytic enzymes and mass spectrometry analysis of the products. The sites of the complex where an antigen binds to the antibody are protected from hydrolysis, and therefore, the comparison of a theoretical map of digestion with the experimental results can provide direct information about the interaction sites. The second procedure, epitope extraction, is based on the formation of a complex between an immobilized antibody and antigen fragments obtained by digestion of the protein in solution; it verifies the excision method results (Bai et al. 1993). The excision/extraction results of epitope mapping of the hCC complex with the mentioned anti-hCC clones, Cyst10 and Cyst28, are summarized in Table 1.

**Table 1:** Comparison of the epitopes identified by epitope extraction/excision and HDX-MS

Antibody	Epitope identified by epitope excision/extraction	Epitope identified by HDX-MS
Cyst10		53–61
	60–70	
	96–102	
	**101**–**111**	**101**–**112**
Cyst28		41–48
	**53**–**62**	**53**–**61**
		65–73
	**85**–**91/92**–**99**	**81**–**99**
	101–111	
NAbs		41–48
	53–62	
		65–73
	92–99	
	101–111	

The use of limited proteolysis of the immobilized antigen–antibody complex and the presence of Sepharose matrix may cause structural changes in the complex components and unspecific binding leading to some false results. Therefore, to verify the epitope sequences obtained with the use of MS-assisted limited proteolysis, epitope mapping was performed by amide hydrogen/deuterium (H/D) exchange in solution coupled with mass spectrometry (HDX-MS). The major advantage of the method is that, in general, in contrast to the previously applied procedure, it is realized in native conditions. In heavy water, the exchange of amide hydrogens at epitopic sites in the antigen-Ab complex is slower due to the changes in the hydrogen bonding status of the backbone amide hydrogens and in their solvent accessibility. Hence, from the comparison of antigen deuteration level at several different time points in the presence and absence of an antibody, the binding site can be localized (Obungu et al. 2013; Brock 2012). Usually, HDX MS findings are verified with other techniques. Nowadays, amide H/D exchange coupled with proteolysis and MS analysis represents a basic tool in studying protein conformation, aggregation processes and protein–protein interactions (Prądzińska et al. 2016).

Herein, we describe the epitope identification in the hCC molecule for two anti-hCC monoclonal antibodies, Cyst10 and Cyst28, by H/D exchange combined with mass spectrometry. In this work, comparison of two well-known mass spectrometry methods applied to identification of interaction sites in antigen–antibody complex is presented. The hCC epitopes recognized by Cyst10 and Cyst28 previously defined by MS-coupled limited proteolysis are of the discontinuous kind. The present results from the HDX-MS approach are mostly in accordance with the epitopic sequences obtained by proteolytic excision/extraction (Śladewska et al. 2011). In addition, we made an attempt to localize with the HDX-MS approach the epitopes for natural, polyclonal anti-hCC autoantibodies (NAbs) isolated from human IgG fraction, and compare them with our previous results obtained using an MS-assisted proteolysis approach (Johnstone and Thorpe 1996).

## Experimental procedures

### Materials

Mouse monoclonal antibodies Cyst10 (5 mg/ml) and Cyst28 (6 mg/ml) were purchased from HyTest Company (Turku, Finland) (4CC1). The protein dilution buffer was phosphate buffer saline (PBS; 5 mM Na_2_HPO_4_, 150 mM NaCl; pH 7.4).

### Human cystatin C expression

The hCC was overexpressed in *E. coli* strain C41(DE3) and purified by ion-exchange chromatography as described previously (Szymańska et al. 2009). The protein purity was characterized by SDS–PAGE, Size Exclusion Chromatography, and Mass Spectrometry (see Supplementary Materials Figure 1).

### Isolation of natural antibodies against human cystatin C (NAbs)

Isolation of NAbs was performed as described previously (Johnstone and Thorpe 1996). Briefly, 25 mg of IgG fraction from human serum was applied onto an hCC-Sepharose column equilibrated in PBS (pH 7.4) and incubated overnight at 4 °C with gentle shaking. After washing with PBS, the affinity–bound antigen–antibody complex was dissociated with 10 × 500 μl of 0.1 % aqueous TFA (pH 2.5). The isolated NAbs were analyzed by SDS–PAGE, and their concentration was determined by measuring the absorbance at 280 nm (NanoQuant, Infinite M200Pro, Tecan) using the extinction coefficient *E*_280_^1%^ = 14 (Johnstone and Thorpe 1996).

### Sample preparation

For epitope mapping, 75 pmol of hCC and Abs were mixed and incubated for 1 min at room temperature. Each hCC–Abs complex sample was compared in the HDX MS experiment to a control sample containing the same amount of hCC and the same ionic composition as the corresponding complex (75 pmol of hCC and PBS instead of an Abs solution). Total reaction volume was 20 µL, while the final deuteration level of the buffer was 83.8 % for Cyst10, 85.6 % for Cyst28, and 71.3 % for NAbs.

Deuteration was performed at room temperature for 1, 5, 10 or 100 min, in quadruplicates. Each reaction was stopped by adding 30 µl of quenching solution (3.34 M urea, 500 mM tris(2-carboxyethyl)phosphine and 23.2 mM KH_2_PO_4_) and flash-freezing in liquid nitrogen. The hCC solution deuterated for 3 days was used as a fully deuterated sample for back-exchange correction.

### Sample analysis: LC MS

Each labeled and quenched sample was analyzed in a semi-automated HDX-MS system (Biomotif AB, Danderyd, Sweden) in which manually injected samples were automatically digested, cleaned and separated at 2 °C. Deuterated samples were digested using a column with immobilized pepsin (2.1 × 30 mm, Applied Bioscience) for 75 s at a 70 µl/min flow protocol, followed by an online desalting step on C-18 precolumn (2 mm I.D × 10 mm, ACE HPLC Columns, Aberdeen, UK) using 0.05 % TFA at a 300 µl/min flow rate for 3 min. Peptic peptides were then separated by a 17 min 10–35 % linear gradient of acetonitrile/water in 0.3 % formic acid using a HALO C18/1.8 µm analytical column (2 mm ID × 50 mm) operating at a 110 µl/min flow rate. An Orbitrap XL mass spectrometer (Thermo Fisher Scientific) operated at 60,000 resolution at *m*/*z* 400 was used for the MS analysis.

Several LC MS/MS runs were carried out to identify the peptides in the hCC pepsin digest. The Mascot software (Matrix Science) was used to search MS/MS data in a database composed of the cystatin sequence using the following parameters: variable modifications—oxidation of methionine; enzyme setting—“none”; peptide and fragment mass tolerances of ±5 ppm and ±0.6 Da, respectively. Peptides with Mascot ion scores higher than 20 were further selected for HDX kinetic studies. In addition, each selected peptide was further validated by manual inspection of the MS/MS spectrum. The HDExaminer software (Sierra Analytics, Modesto, USA) was used to process all HDX-MS data.

### Results peptic peptides of human cystatin C: HDX experiment

**Fig. 2:**
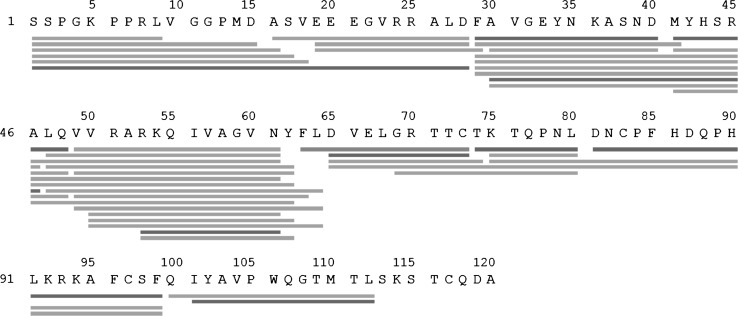
Peptides detected by LC–MS after pepsin digestion of human cystatin C. *Red lines* indicate peptides for which deuteration level analysis was performed (Figs. 4, 5, 6). *Green lines* indicate other detected peptides

To assess the effect of the antibody binding to human cystatin C, HDX-MS analysis of the monomeric protein was performed. Unlabeled hCC was subjected to online pepsin digestion, desalting, chromatography, and tandem mass spectrometry analysis. To achieve high sequence coverage of peptides obtained after enzymatic digestion with pepsin, various digestion conditions (different denaturing reagents, variable enzyme: protein molar ratio) were tested. It was found that enzymatic digestion carried out in solution on ice was not effective enough. Therefore, digestion of the protein on the column was attempted. This experiment resulted in a sequence coverage of 93 % (43 peptic peptides presented in Fig. 2). From the digestion of the N-terminal fragment of human cystatin C, 9 fragments were obtained. The shortest of them had 9 amino acid (AA) residues, and the longest one—28 AA residues. The majority of the peptides were about 15-AA long. The central part of the protein (29–64) was the most efficiently digested. Looking on the primary cystatin C structure (Fig. 2), one can notice that one of the digestion sites is located around residues 28/29, i.e., in the central part of the α-helix (Fig. 1). However, some of the obtained digestion fragments were longer than 20 amino acid residues and covered the second beta strand (β2) and loop 1 (L1) (Fig. 1). A fragment of the protein from residues 65–99 was digested with formation of only nine peptides. Structurally, this hCC region represents part of the β3 strand and an appendix structure (AS). The 100–112 fragment, covered by 3 peptides, represents β4 strand, loop 2 (L2) and part of the last, β5, strand. Surprisingly, the C-terminal fragment of the protein was not detected in any of the performed experiments. As similar problems were encountered in our other experiments, it is possible that the lack of C-terminal sequences in peptic mixtures is related to difficult ionization of the former. All peptides obtained after digestion and MS-analyzed with deuteration levels determined are shown in Figs. 4, 5 and 6. In Fig. 2, only their shortest common fragments are shown (red lines).

### Epitope identification

**Fig. 3:**
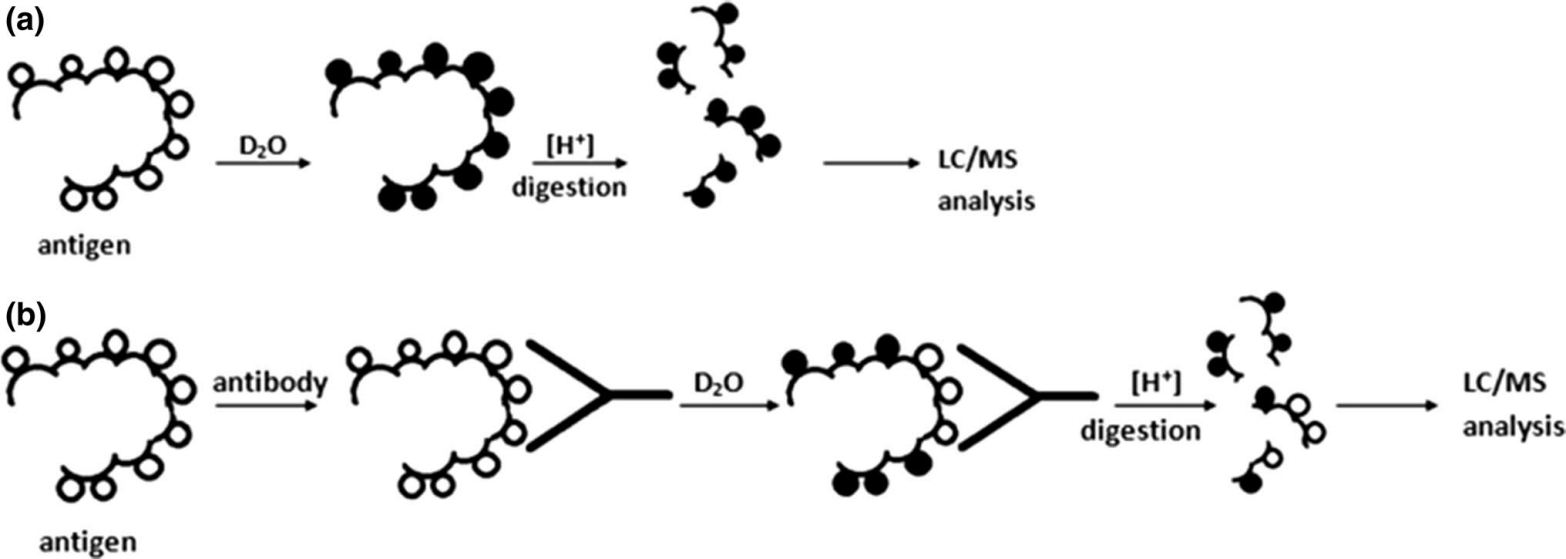
Scheme of the on-exchange analysis approach for the epitope mapping of the hCC–Cyst10/28/NAbs complexes by HDX-MS: **a** control experiment; **b** sample analysis

The hydrogen–deuterium exchange experiments were performed on the free antigen as a reference (control) experiment and on the antigen–antibody complexes. In samples containing the antigen–antibody complex, human cystatin C was mixed with one of the antibodies (Cyst10, Cyst28 or NAbs) and with the deuterated buffer. In the control samples, human cystatin C was mixed with PBS instead of the Ab solution (the same volume) and with the deuterated buffer. The samples were incubated for four different periods of time and quenched (see Sample Preparation and Fig. 3). Then, the samples were digested on the pepsin column and analyzed in a semi-automated HDX-MS system. Undeuterated hCC was also digested on the pepsin column, and peptic peptides were analyzed by LC–MS/MS. To determine the maximal deuterium incorporation properly, hCC was deuterated for 3 days and analyzed as other samples. To process the HDX-MS data, HDExaminer software (Sierra Analytics, Modesto, USA) was used. For individual peptides, deuteration levels obtained after digestion of free hCC and hCC involved in the complex with the antibody were compared. For some protein fragments, maximal deuterium incorporation was reached very quickly. The N-terminal fragment, with its disordered secondary structure, was an example of such a region. This fragment reached circa 100 % deuteration after a 1-min incubation in the deuterated buffer on ice (data not shown).

#### Epitope location for the Cyst10 antibody

**Fig. 4:**
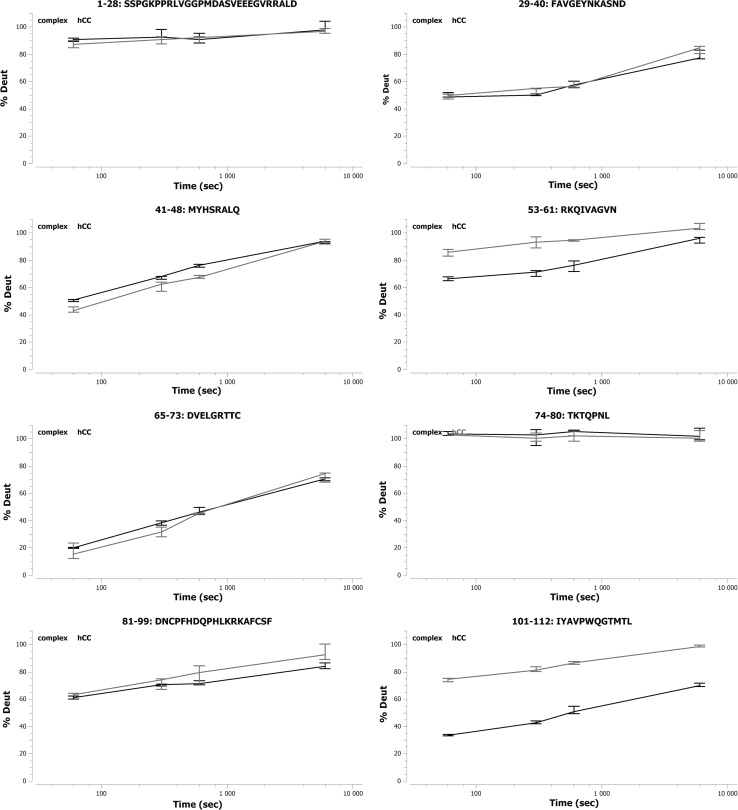
Deuteration level of the analyzed fragments of hCC in the presence (*black color*) and in the absence (*blue color*) of Cyst10 antibody

HDX-MS experiments showed that the epitope for the antibody Cyst10 in human cystatin C is discontinuous and located in the middle of the molecule as well as in the C-terminal part. Two peptides (53–61 and 101–112) out of eight hCC fragments obtained after digestion demonstrated statistically significant differences in the deuteration levels in the absence of the Ab and upon the antibody binding (Figure 4). The C-terminal fragment, 101–112, showed bigger difference (15–40 % on average) than the fragment 53–61 located in the middle part of the protein (15–20 % on average). Other peptides from these regions partially overlapping the sequences 53–61 and 101–112 also showed large differences in the level of deuteration (see Supplementary Materials, Figure S2).

#### Epitope location for the Cyst28 antibody

**Fig. 5:**
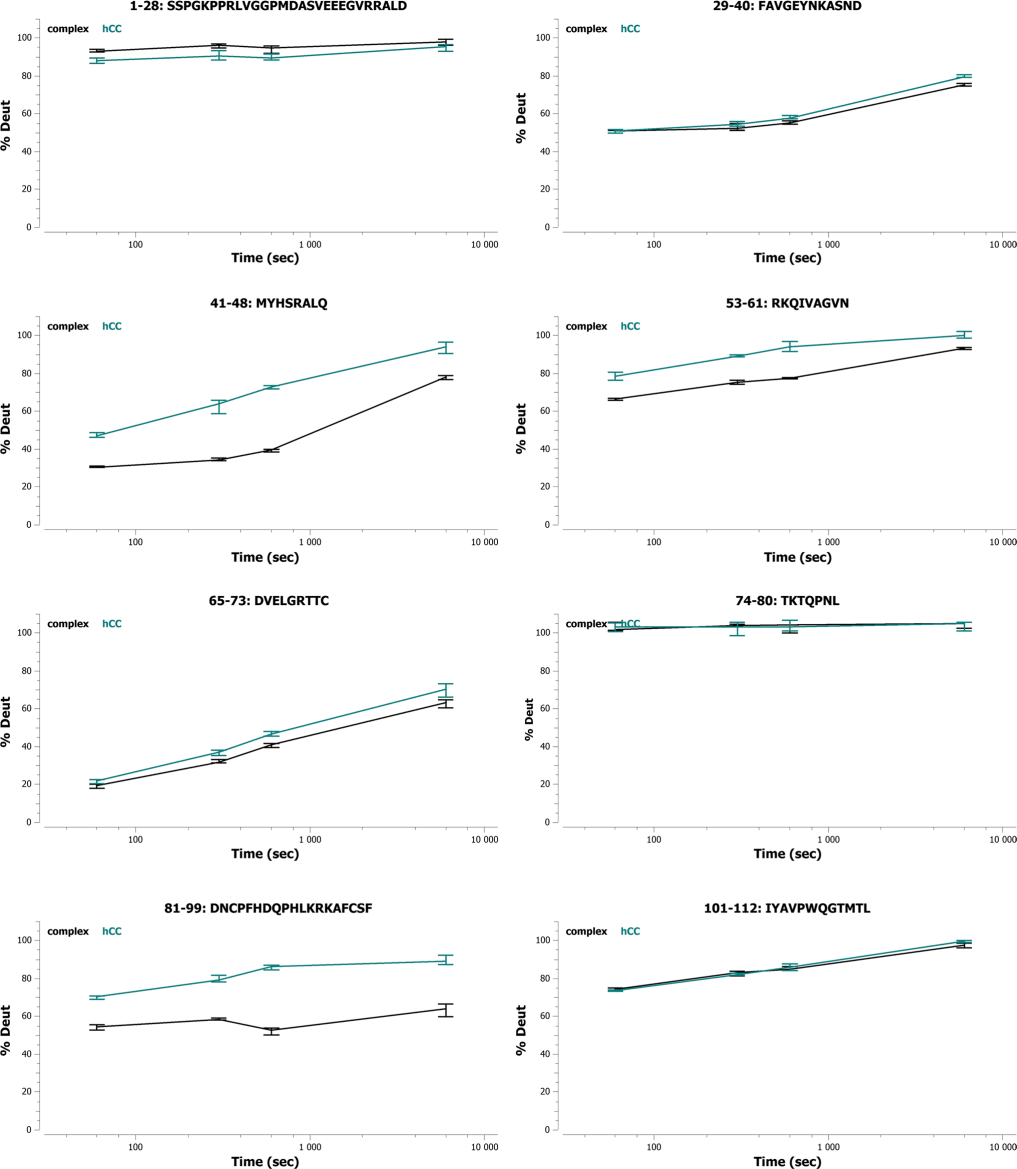
Deuteration level of the analyzed fragments of hCC in the presence (*black color*) and in the absence (*blue color*) of the Cyst28 antibody

Three short (41–48, 53–61, and 65–73) and one longer (81–99) fragment of hCC showed statistically significant differences in deuteration levels between the presence and the absence of the mAb (Figure 5, and Supplementary Materials Figure S3). In the case of peptides 81–99 and 41–48, the deuteration level of hCC in the presence of mAb was much lower (by approximately 20–30 %) than in the absence of Cyst28. However, for the fragments 53–61 and 65–73, much lower deuteration level differences were observed (15 and 5 % on average, respectively). According to the HDX-MS results, the mentioned fragments of hCC seem to be the epitopic sequences for the Cyst28 antibody.

#### Epitope location for NAbs

**Fig. 6:**
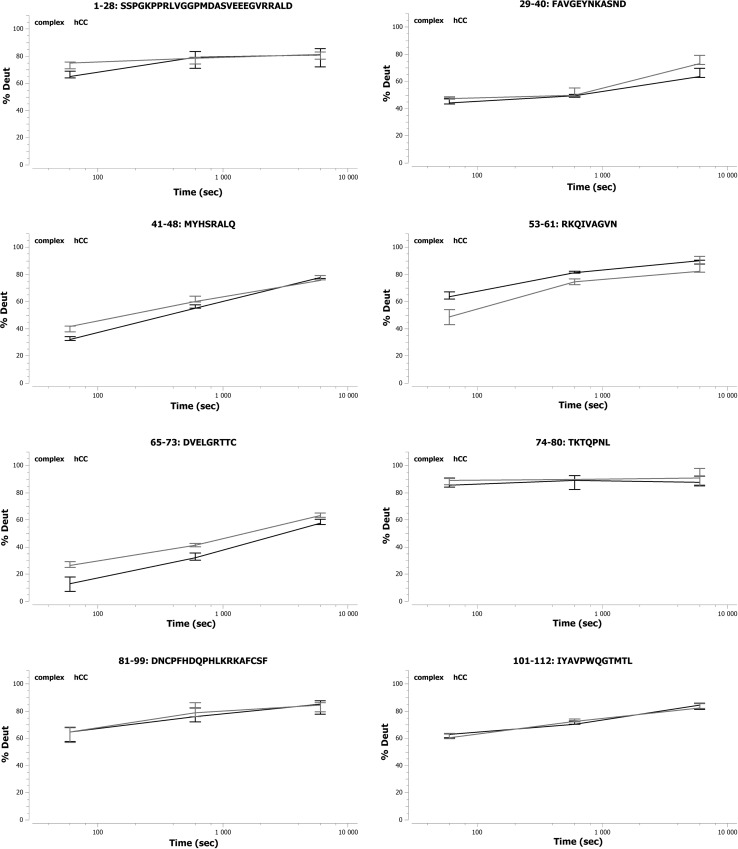
Deuteration level of the analyzed fragments of hCC in the presence (*black color*) and the absence (*blue color*) of NAbs

Two peptides (41–48 and 65–73) demonstrated statistically significant differences in the deuteration level between the presence and the absence of NAbs (Fig. 6; Supplementary Materials Figure S4). For the peptide 41–48, these differences (<10 %) were observed only at two time points (1 and 10 min).

## Discussion

To get deeper knowledge about the molecular mechanism of human cystatin C dimerization process, it may be important to identify sites of hCC interaction with anti-cystatin C antibodies able to suppress formation of hCC dimers. The amino acid sequences and the localization of the short epitopic sequences within the hCC molecule could be useful for better understanding of fundamental for hCC oligomerization domain swapping process (Janowski et al. 2001; Wei et al. 2014), and for design of small inhibitors or nanobodies for future immunotherapies. In this study, HDX-MS was used to identify the epitopes for two monoclonal antibodies, Cyst10 and Cyst28, as well as natural autoantibodies NAbs.

The H/D exchange MS method was chosen as it is performed at native conditions and is able to map conformational epitopes, while the previously applied extraction/excision MS method is more complicated and less reliable due to possible conformational changes accompanying immobilization and, as well as the fact that tryptic digestion may perturb the higher order structure of the antigen. Nevertheless, it should be remembered that HDX-MS method has its own limitations. One of the problems which can appear at the stage of immunocomplex formation is occlusion of solvent molecules between the two partners. Such an event can reduce the exchange rate (steric exclusion of solvent) (Jaskólski 2001). Another thing that can cause a problem is the formation of new interactions between the antigen and antibody, which can stabilize exchangeable amide hydrogens. To the important factors that may introduce additional complications, we can add the change in the structure of protein in the regions distant from the epitope, resulting from the allosteric effects. Furthermore, the enhanced thermodynamic stability of the protein (the result of complex formation) can reduce the HDX rate. Therefore, it is reasonable to use more than one method for the epitopic sequence identification, and in our case, we present the comparison of epitope extraction/excision MS with the HDX MS method.

**Fig. 7:**
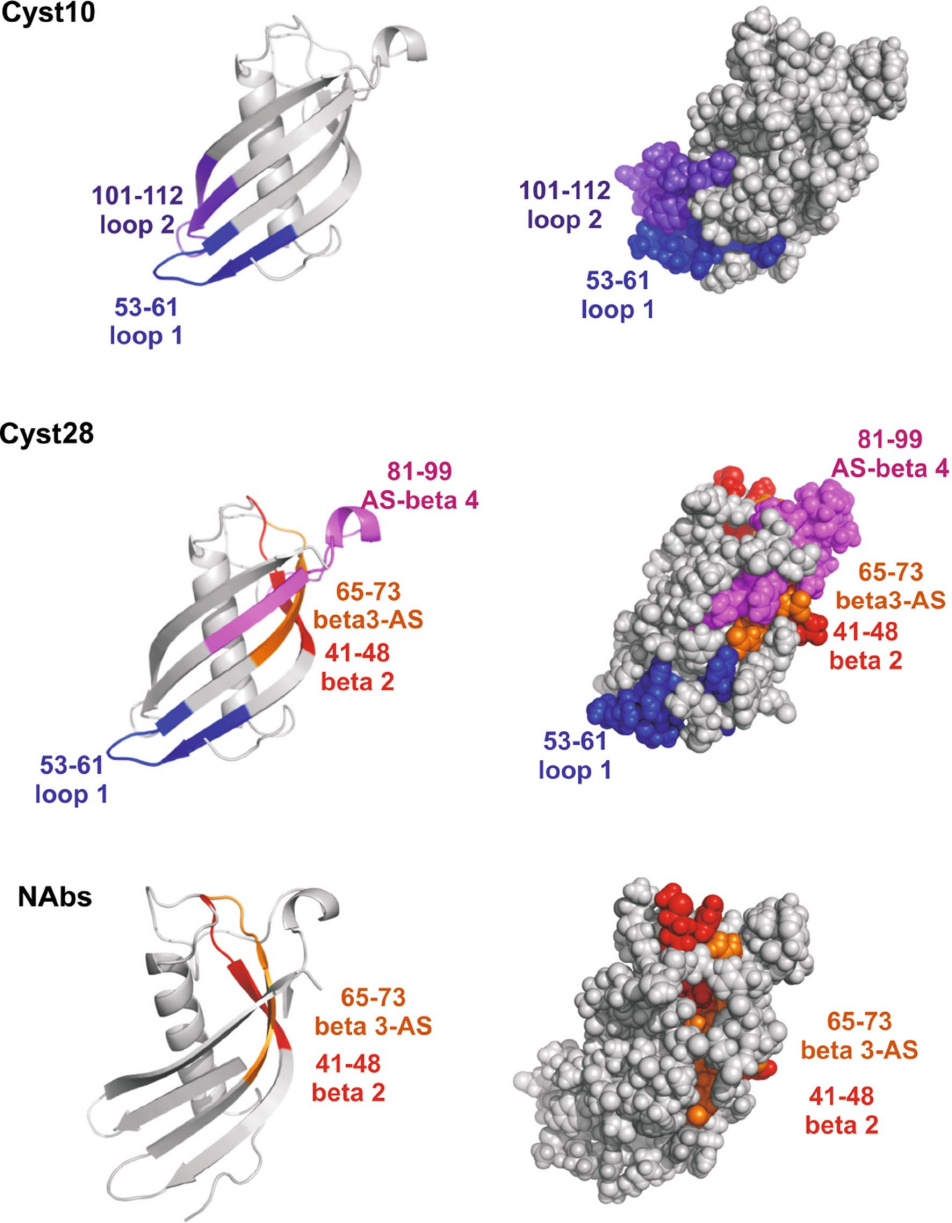
Graphic representation of epitope locations for the studied antibodies

The choice of two monoclonal antibodies Cyst10 and Cyst28 may be of special importance as they possess opposite inhibitory properties toward the dimerization of human cystatin C. HDX-MS experiments confirmed our earlier results (Śladewska et al. 2011), indicating that the epitopes for both antibodies are of discontinuous type and are located in the middle part as well as in the C-terminal part of the cystatin C molecule. It was found that the Cyst10 antibody recognizes two fragments which are located in two loops of the hCC structure (53–61—loop L1, 101–112—loop L2, see Figs. 1, 7). It is possible that the epitope for the antibody Cyst28 is split into four fragments. Three fragments are located within β-strands: 41–48 in β2, 65–73 in β3, and 81–99 in β4, while the fragment 53–61 covers the sequence of the loop L1 (see Fig. 7).

In our previous report, identification of the epitopes for the same monoclonal antibodies with the use of epitope excision and extraction procedures combined with mass spectrometry was presented (Śladewska et al. 2011). The comparison of epitopes identified by two methods, excision/extraction of epitope and HDX-MS, is given in Table 1.

For the Cyst10 antibody, the fragment corresponding to the sequence of loop L2 (101–112) was identified using both methods, and in the ELISA experiment, the fragment presented the highest affinity to the Cyst10 clone (Śladewska et al. 2011). The differences between the epitopic sequence locations found using both methods exist in the fragment located in the middle of human cystatin C. The fragment 60–70, identified in the extraction/excision experiments, is a part of the β3-strand, while the 53–61 fragment, localized using the HDX-MS approach, covers the amino acids sequence in loop L1. Digestion of hCC by pepsin does not generate a fragment corresponding to the sequence 96–102. In our previous studies, two fragments (60–70 and 96–102) displayed weak affinity to Cyst10 in the ELISA experiments (Śladewska et al. 2011). In the case of the antibody Cyst28, both methods indicate that a part of the epitope is localized within loop L1, and covers residues 53–61. Similarly, the hCC fragment from the C-terminus (81–99) revealed in the epitope excision/extraction procedure, was confirmed in HDX-MS experiments to be a part of the hCC interacting with Cyst28 sequence. In the excision/extraction method, this sequence was identified as two separate tryptic peptides: 85–91 and 92–99, whereas in HDX-MS experiments, a longer peptic fragment covering this region (81–99) was identified and analyzed. The fragment 92–99, identified in the excision/extraction method, revealed the highest affinity to clone 28, while the fragment 85–91 revealed moderate affinity to that antibody in the ELISA test (Śladewska et al. 2011). Two cystatin C fragments (41–48 and 65–73) showed in HDX-MS experiments significant differences in the level of deuteration for the free and Cyst28-complexed hCC, and were, therefore, assigned as probable binding sites in the hCC–Cyst28 complex. However, these sequences were not identified in the previously used method as parts of the epitope. The first sequence covers the region from the end of the α-helix to the half of β2-strand of the protein, the residues 65–73 cover β3 and a part of the AS structure. In the case of C-terminus of hCC, which was identified as a binding site by the extraction/excision method, there were no statistically significant differences in the HDX-MS experiments in deuterium incorporation to the fragment 101–112, suggesting that this fragment does not belong to the epitope.

Cystatin C dimerization process requires partial opening of two monomeric structures and exchanging N-terminal domains (β1, α1, β2, L1), which results in formation of a new β-strand (Staniforth et al. 2001). The formation of a new β-structure makes that dimer more stable than the monomeric form (Staniforth et al. 2001). During domain swapping process, conformational changes concerning the flexible loops as well as β-turns occur (Szymańska et al. 2009). The part of the protein that is significantly changed during dimerization is loop L1 which disappears in the dimeric hCC structure (Rodziewicz-Motowidło et al. 2009). This region, with its lack of conformational stability, is considered to be the molecular “spring” facilitating the domain exchange (Rodziewicz-Motowidło et al. 2009).

With the use of HDX-MS methodology, we confirmed that the most important sequences for the Cyst28 antibody binding are located in three β-strands and loop L1. It seems that blocking L1 and separating the β2 strand from the rest of β-strands might be a key element in the dimerization process inhibition. Binding of the antibody to L1 can exert the stabilization effect on the structure of hCC, rendering the domain exchange simply impossible. This hypothesis would explain the data published by the Swedish group, according to which dimerization of cystatin C was reduced up to 75 % in the presence of mAb Cyst28 (Östner et al. 2011).

For the second antibody, Cyst10, the epitope established by the HDX-MS approach is located in both loops, L1 and L2. This result is a little bit surprising, as the crucial L1 loop sequence seemed not to be involved in the antibody binding according to the MS-assisted excision/extraction procedures, making the most striking difference observed for the studied antibodies (Śladewska et al. 2011). It is possible that effective interactions with the flexible hinge loop L1 is not the only factor for inhibition of dimerization by hCC-directed monoclonal antibodies and that supplementary interactions of Cyst28 with other epitopic sequences, e.g., β2-strand (epitopic fragment 41–48) are responsible for the hCC monomer stabilization and dimerization suppression. An additional factor which may have an influence on the very different propensity of both antibodies for hCC dimerization suppression can be related to their different affinity to hCC. It was demonstrated previously using microscale thermophoresis that antibody Cyst28 interacts with hCC ca. six times stronger than Cyst10 (Cyst28—*K*_d_ = 20.2 ± 1.9 nM; Cyst10—*K*_d_ = 141 ± 13 nM) (Śladewska et al. 2011), and that for Cyst10 in aggregational conditions, the domain exchange process is favored in competition with the antibody binding.

In the case of the studied autoantibodies, the search for epitopic hCC fragments by HDX-MS was not fully successful, as it was difficult to notice even smallest differences in deuteration levels of some peptides. Slight differences in the deuterium incorporation between free hCC and hCC in the complex with NAbs can arise from several facts. NAbs are a mixture of different antibodies recognizing different epitopes. Therefore, the determined level of deuteration is an averaged value for the protein associated with various ligands. Nabs, as polyclonal antibodies, interact weaker with the antigen than best monoclonal antibodies. Nevertheless, it was possible to notice differences in the deuteration level for the fragments 41–48 (β2), and 65–73 (β3).

## Conclusions

The hydrogen–deuterium exchange methodology coupled with mass spectrometry is an excellent technique for studying protein–protein interactions, especially when used for epitope identification. Using the HDX-MS method, we identified the location of epitopes for two monoclonal antibodies against human cystatin C and verified the results obtained previously. The epitopes for both antibodies are of discontinuous type. For clone 28, the epitopic sequences are located between the residues 41–48, 53–61, 65–73, and 81–99. HDX-MS experiments confirmed two epitopic fragments determined in our previous studies (53–62 and 85–91/92–99). For clone 10, one sequence was confirmed by both techniques: 101–111 (C-terminus of hCC), while the sequence 53–61 (L1) identified by HDX-MS was not found in our previous studies. It is interesting that both antibodies partially sharing epitope sequences exhibit very different anti-aggregational properties. Moreover, it seems that both Abs interact with the crucial for the dimerization process fragment 55–60 (loop L1). In the previous report on epitope excision/extraction (Östner et al. 2011), we have rationalized the observed impact of the studied antibodies on the dimerization of cystatin C by making an assumption that high dimerization suppression propensity might be related to the antibody Cyst28 interaction with the crucial L1 fragment preventing the hinge fragment from opening, which in turn prevents subsequent association of the protein into dimeric and possibly oligomeric forms. Finding of the 51–63 fragments among the potential epitopic sequences also for Cyst10 suggests that very low dimerization suppression potency observed for clone 10 may also be related to other factors (see “Discussion”).

In the case of polyclonal autoantibodies, we have not found any common sequences identified by both methods. HDX-MS identified two new fragments that can be considered as potential epitopes: 41–48 and 65–73. Significant differences between the results of both methods and the lack of evident epitopic fragments (especially in the C-terminal part of hCC) may arise from weaker interactions between these polyclonal antibodies and the antigen. Overall, the data obtained with HDX-MS shed new light on the epitopes location in human cystatin C, what may be important for future design of hCC dimerization inhibitors.

## Electronic supplementary material

Below is the link to the electronic supplementary material.

Figure S1 MALDI intact mass spectrum of human cystatin C (TIFF 1460 kb)

Figure S2 Deuteration level of the hCC fragments in the presence (black color) and in the absence (blue color) of Cyst10 antibody (PDF 1541 kb)

Figure S3 Deuteration level of the hCC fragments in the presence (black color) and in the absence (blue color) of Cyst28 antibody (PDF 3034 kb)

Figure S4 Deuteration level of the hCC fragments in the presence (black color) and in the absence (blue color) of polyclonal NAbs antibody (PDF 1674 kb)

Supplementary material 5 (DOCX 17 kb)

## Abbreviations

H/D: Hydrogen/deuterium
HDX MS: Hydrogen–deuterium exchange mass spectrometry
hCC: Human cystatin C
HCCAA: Hereditary cystatin C amyloid angiopathy
AD: Alzheimer’s disease
NAbs: Autoantibodies
AS: Appending structure
