# Supplementary figures and images for: Application of amide hydrogen/deuterium exchange mass spectrometry for epitope mapping in human cystatin C
Source: Amino Acids. 2016 Aug 29;48(12):2809–20. doi: 10.1007/s00726-016-2316-y (PMC5107209; doi:10.1007/s00726-016-2316-y)

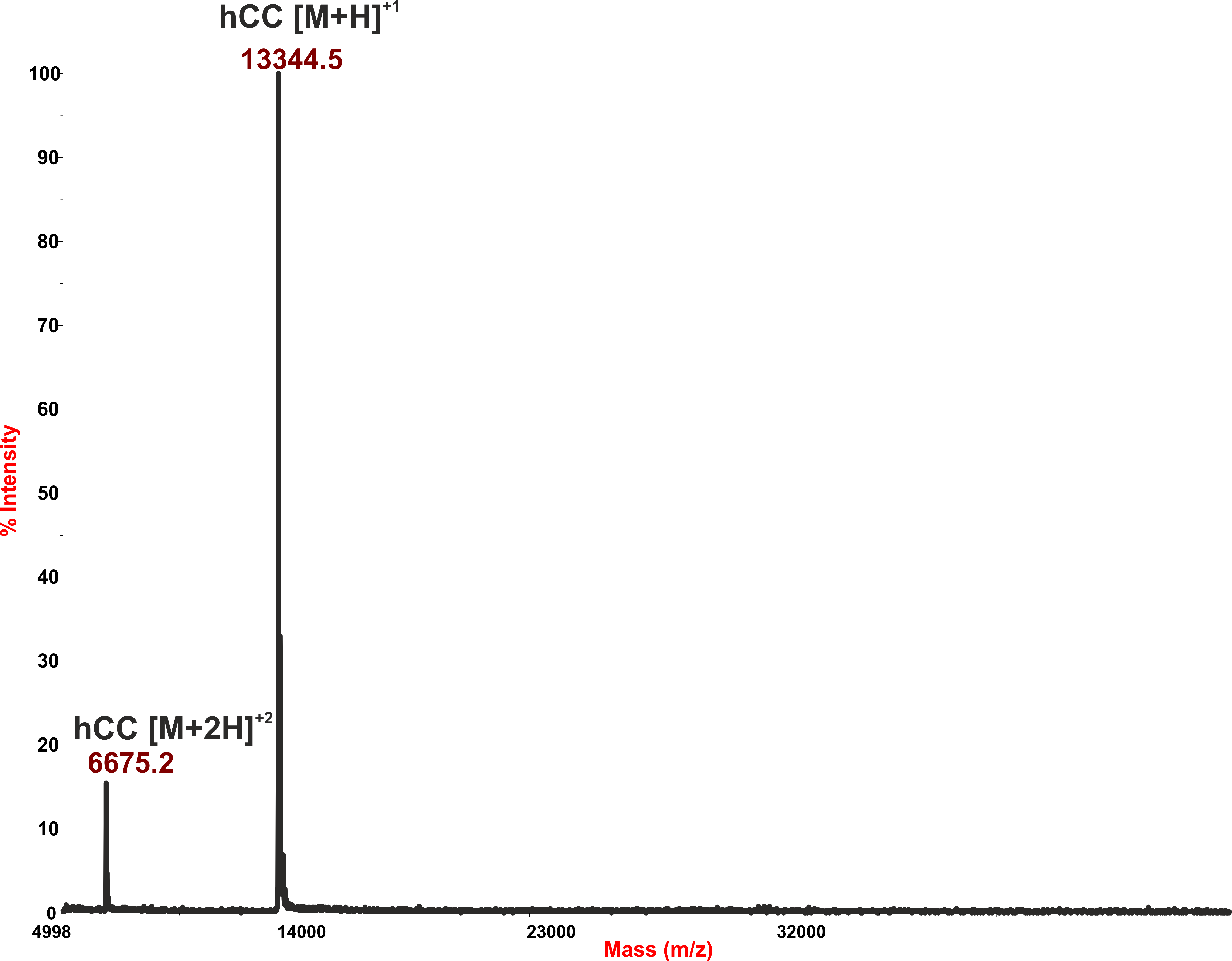

Supplement: Supplementary file 1 — Figure S1 MALDI intact mass spectrum of human cystatin C (TIFF 1460 kb) [file 726_2016_2316_MOESM1_ESM.tif]

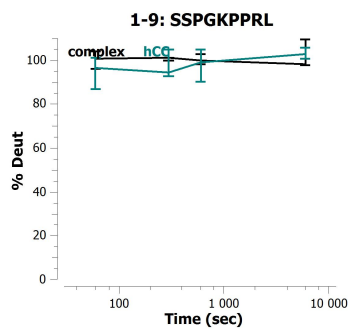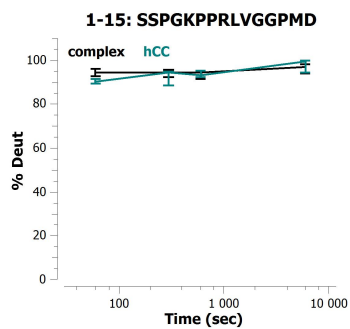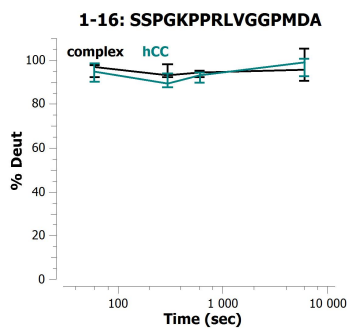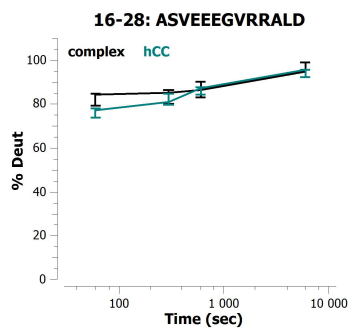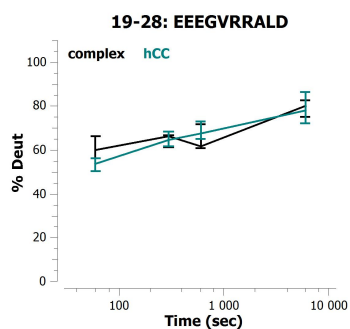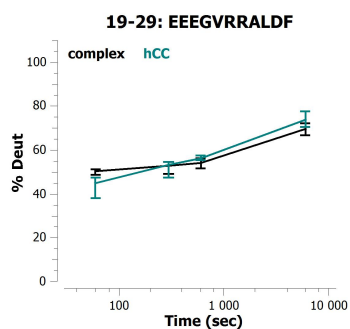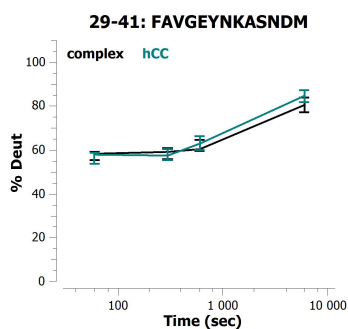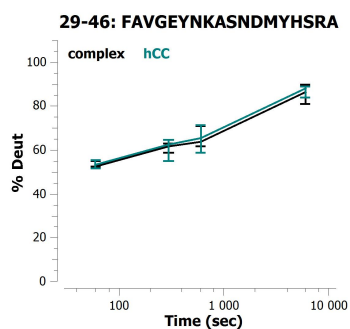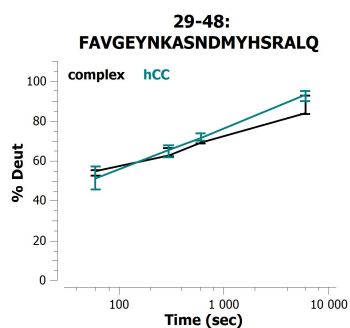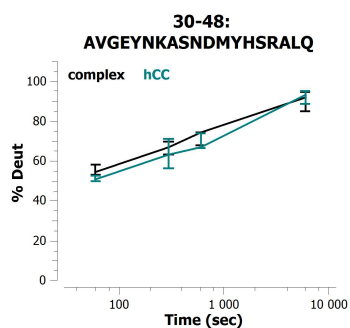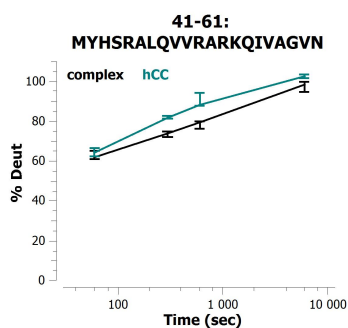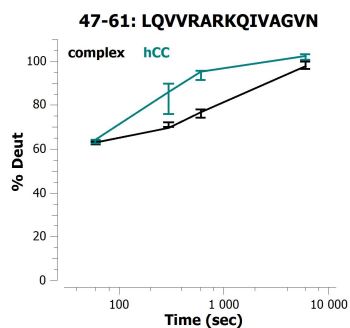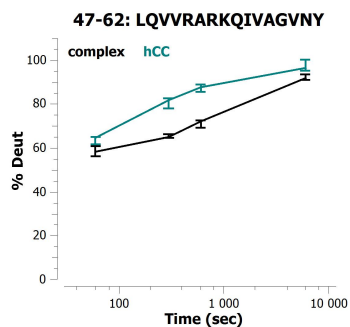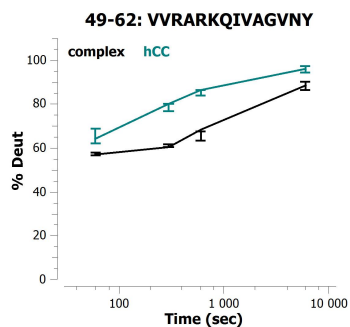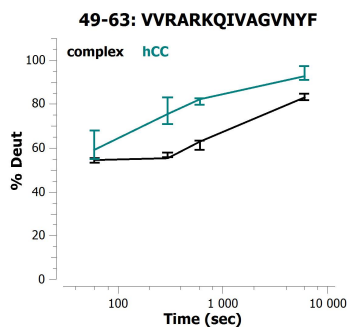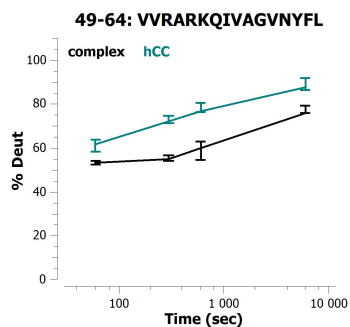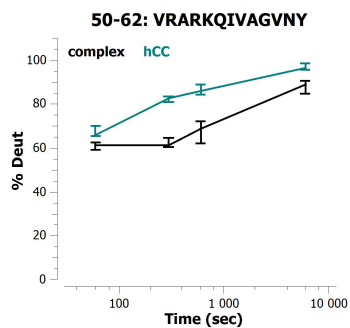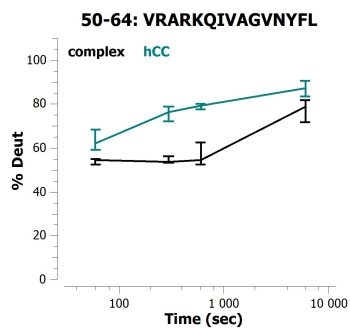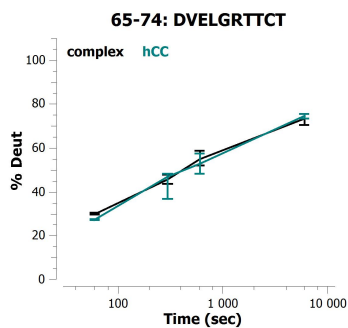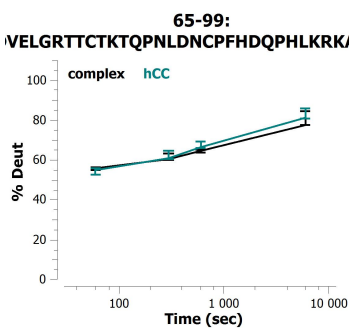

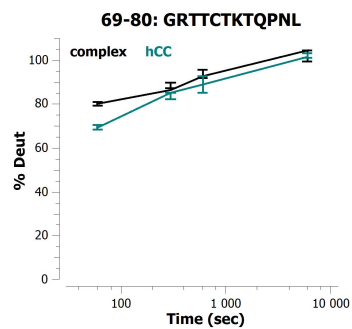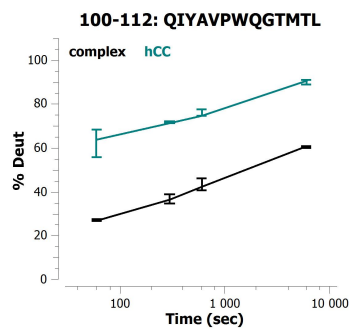

Supplement: Supplementary file 2 — Figure S2 Deuteration level of the hCC fragments in the presence (black color) and in the absence (blue color) of Cyst10 antibody (PDF 1541 kb) [file 726_2016_2316_MOESM2_ESM.pdf]

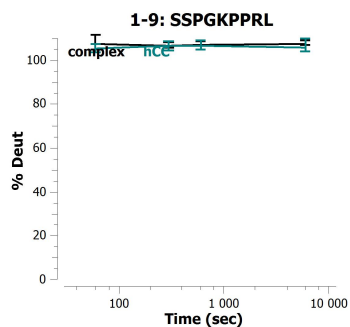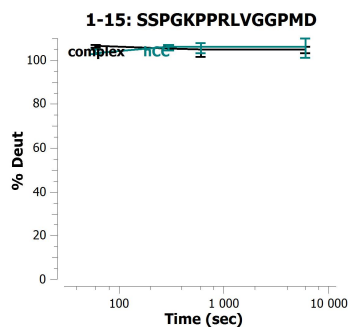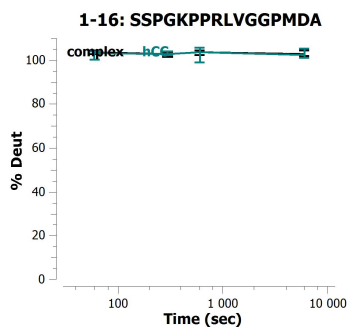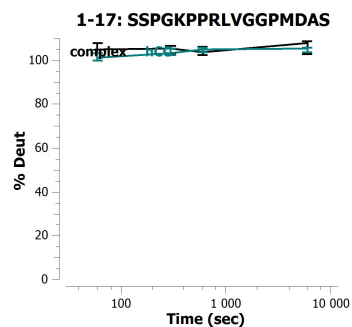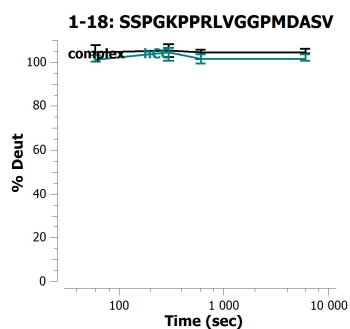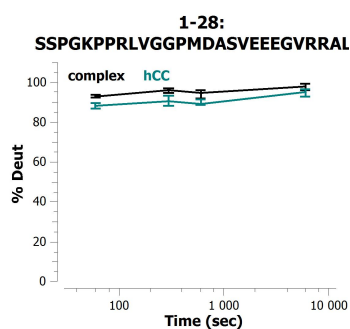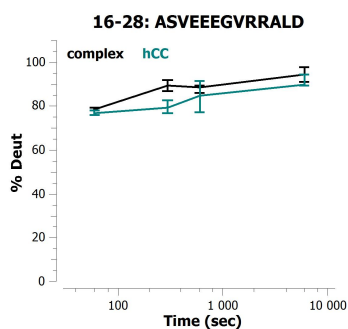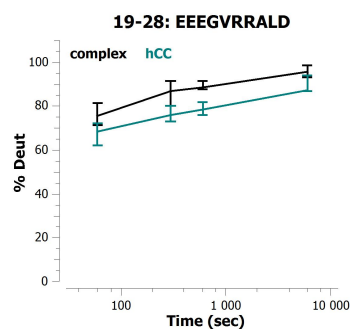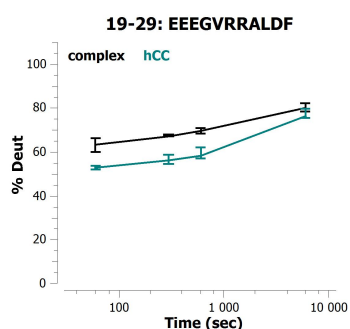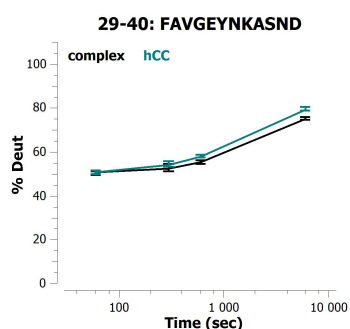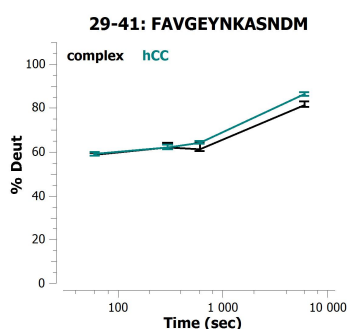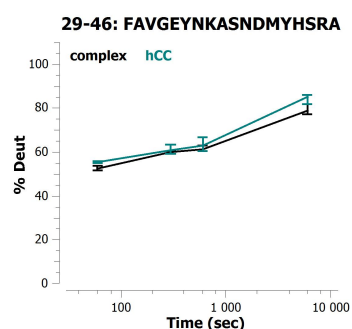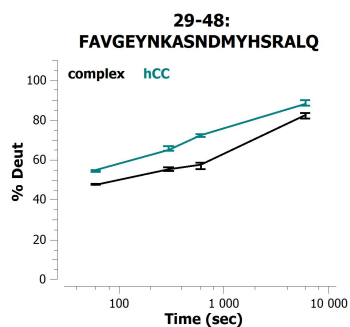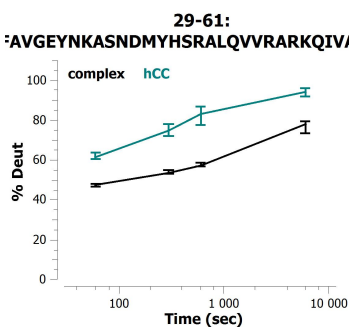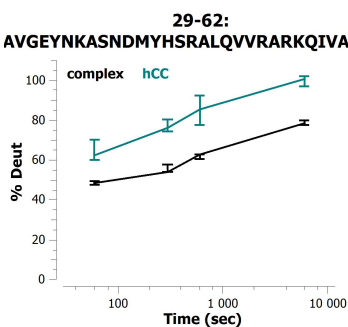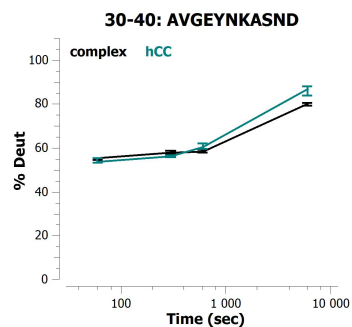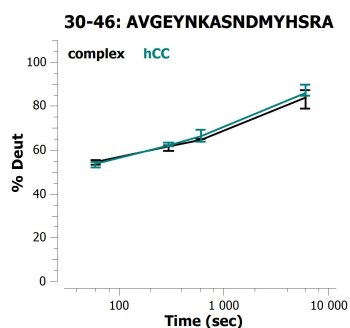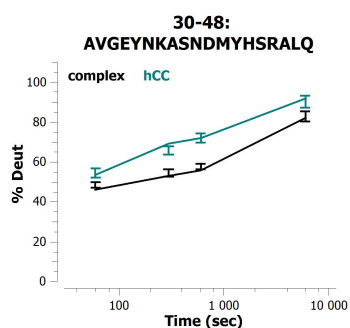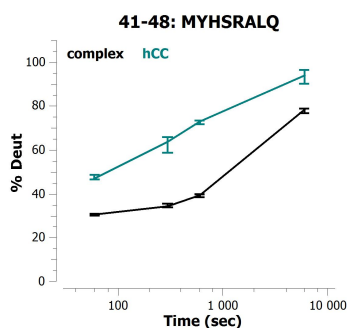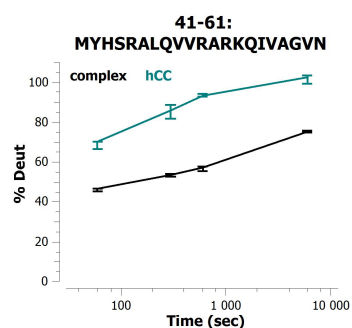

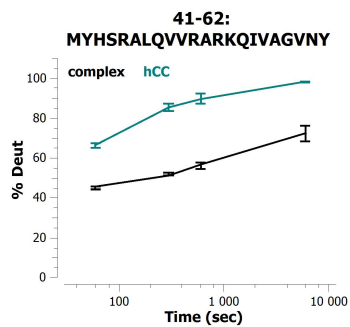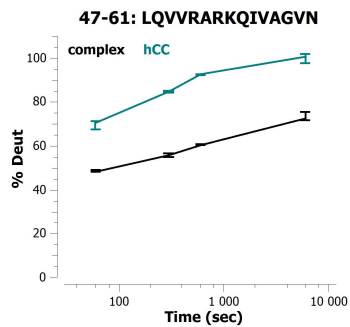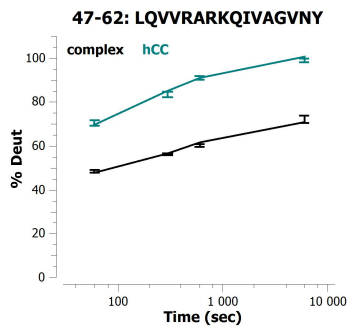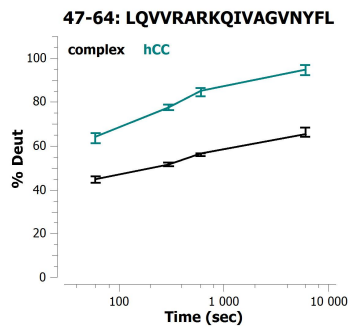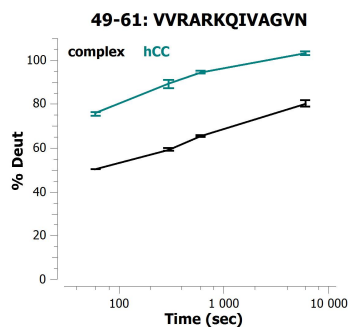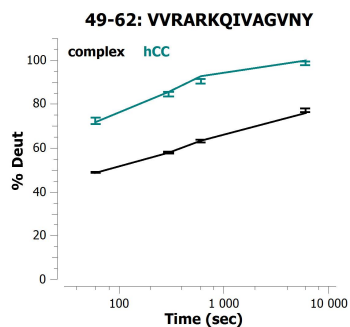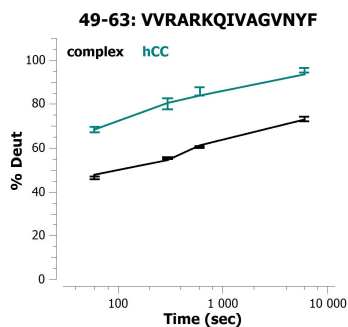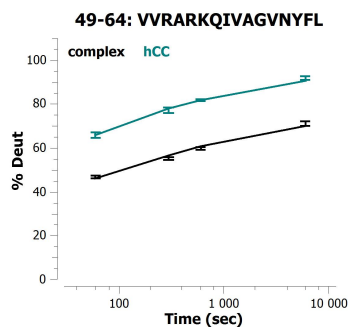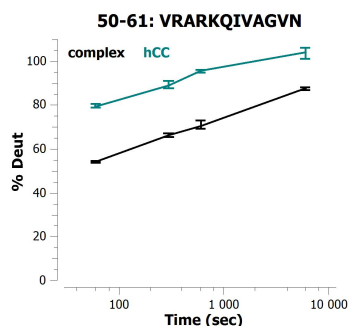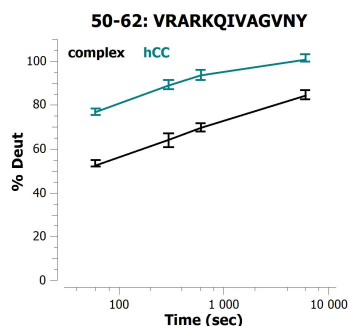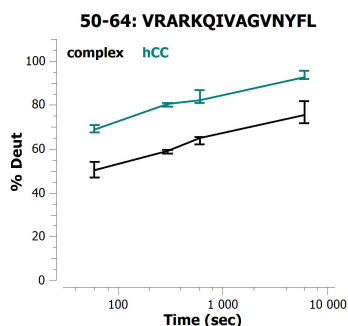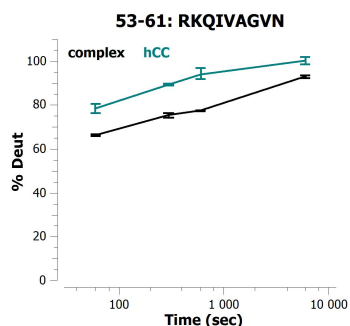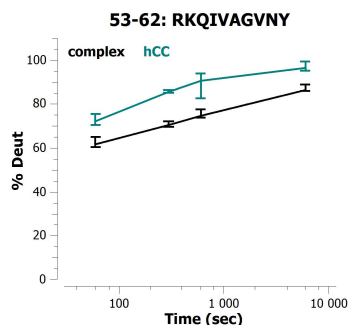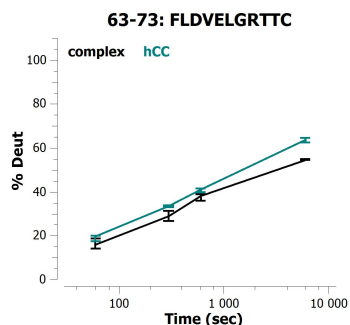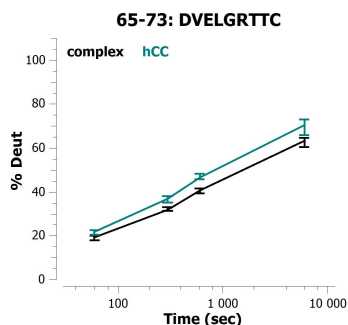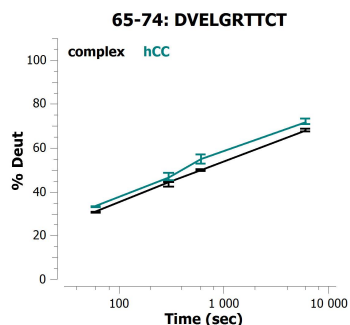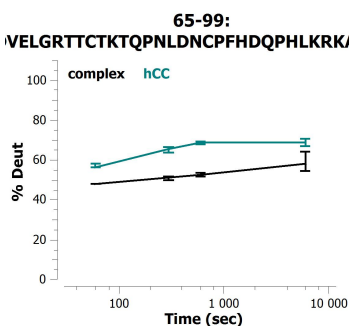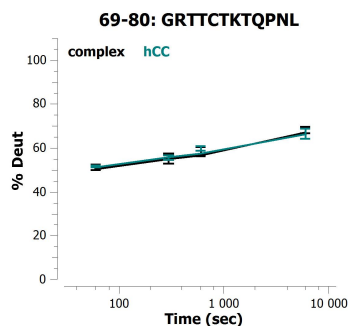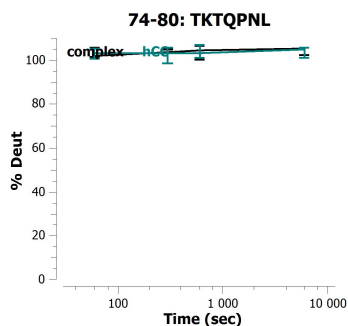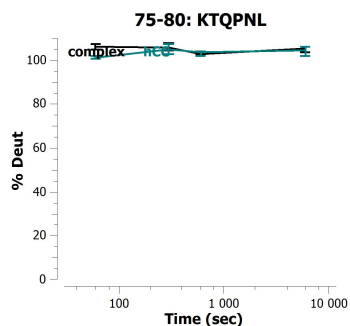

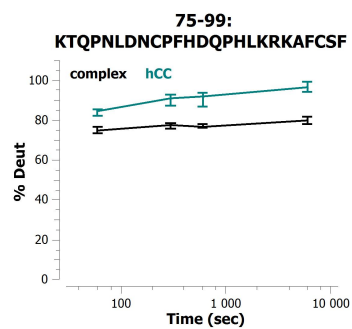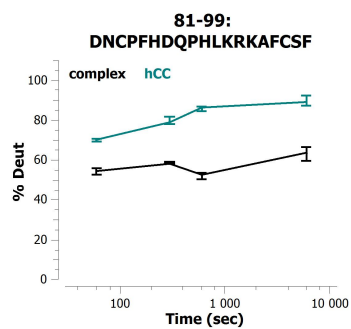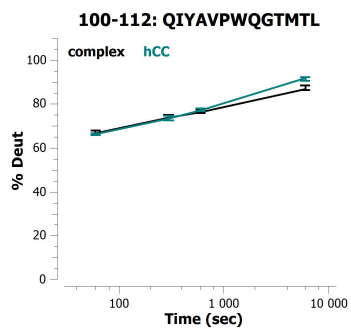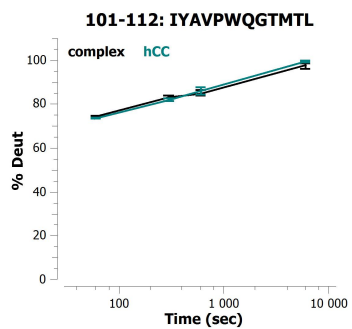

Supplement: Supplementary file 3 — Figure S3 Deuteration level of the hCC fragments in the presence (black color) and in the absence (blue color) of Cyst28 antibody (PDF 3034 kb) [file 726_2016_2316_MOESM3_ESM.pdf]

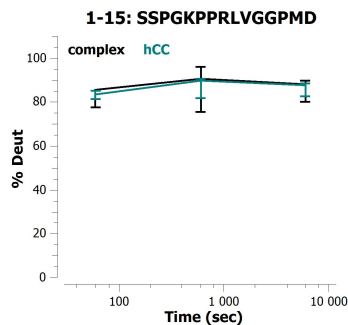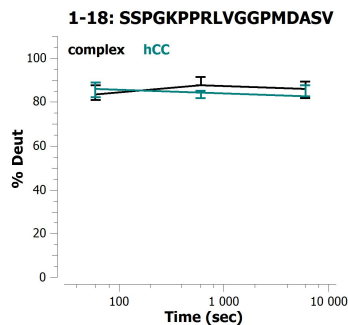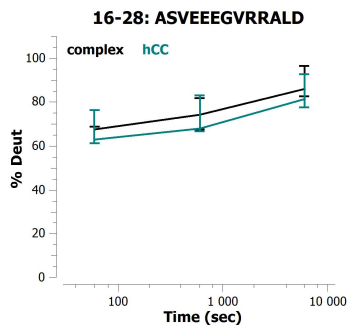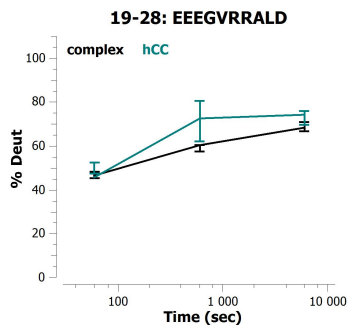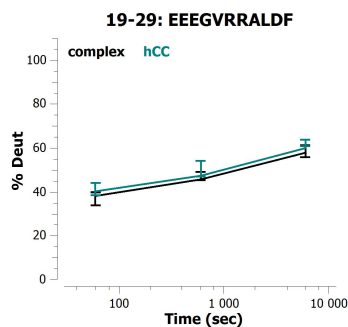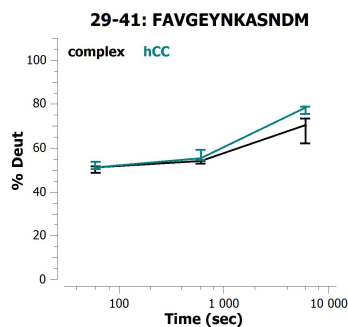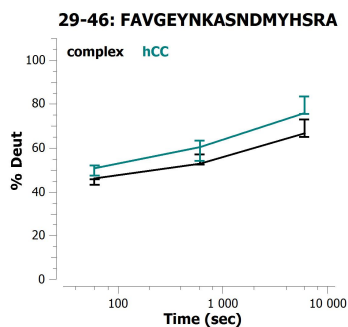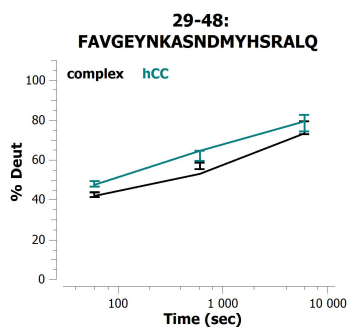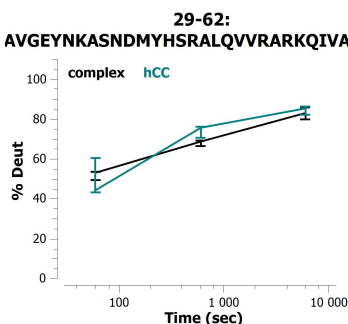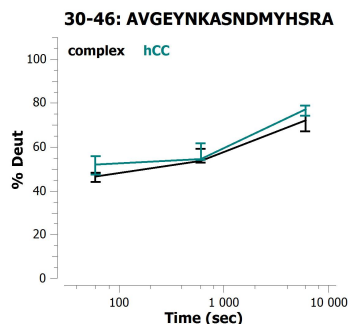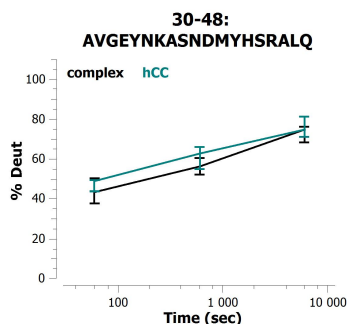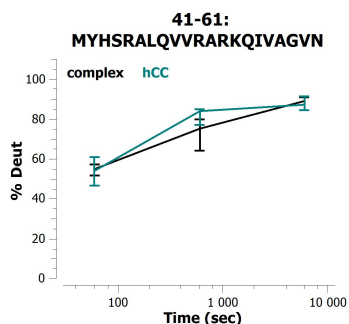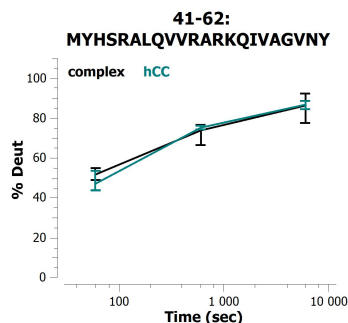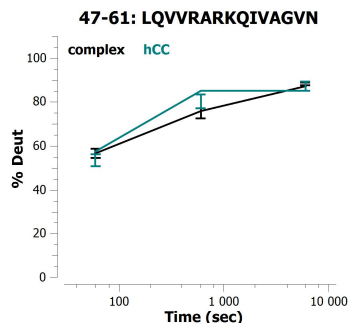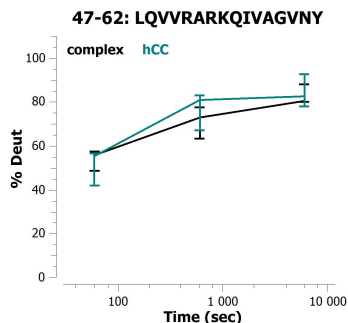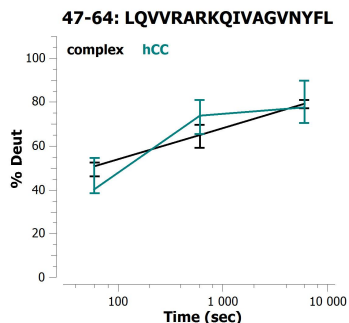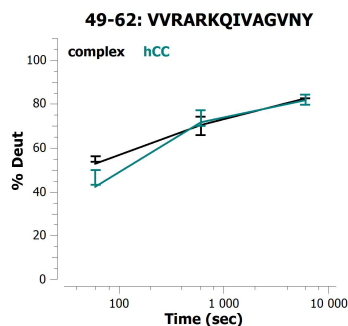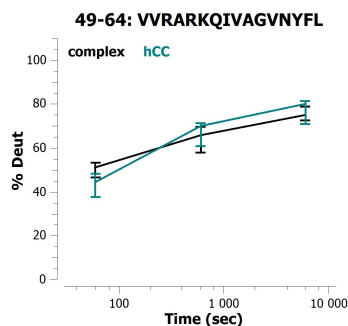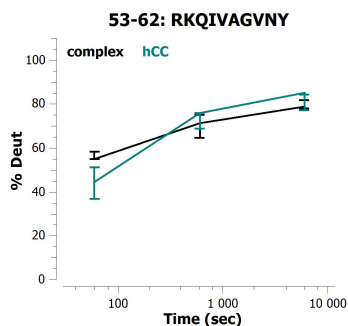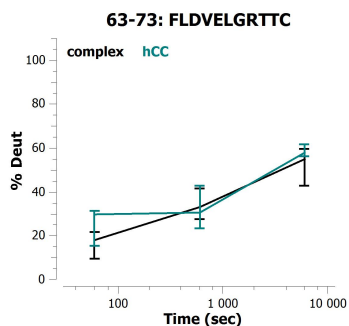

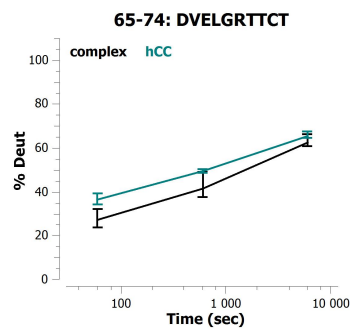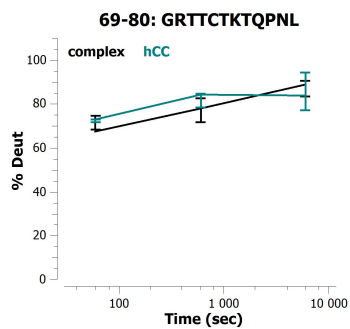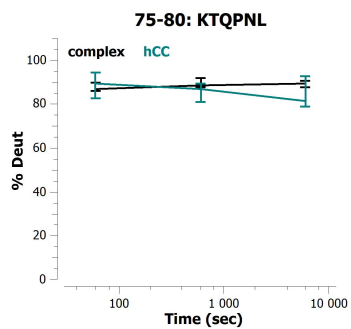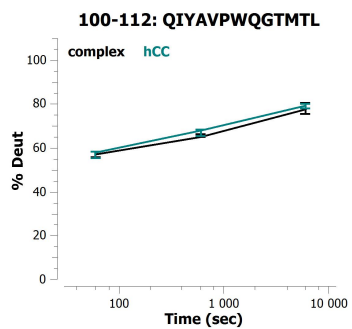

Supplement: Supplementary file 4 — Figure S4 Deuteration level of the hCC fragments in the presence (black color) and in the absence (blue color) of polyclonal NAbs antibody (PDF 1674 kb) [file 726_2016_2316_MOESM4_ESM.pdf]
