# Supplementary material for: Application of amide hydrogen/deuterium exchange mass spectrometry for epitope mapping in human cystatin C
Source: Amino Acids. 2016 Aug 29;48(12):2809–20. doi: 10.1007/s00726-016-2316-y (PMC5107209; doi:10.1007/s00726-016-2316-y)
Supplement: Supplementary file 5 — Supplementary material 5 (DOCX 17 kb) [file 726_2016_2316_MOESM5_ESM.docx]

Supplementary materials:

Figure S1 *MALDI intact mass spectrum of human cystatin C.*

Figure S2 *Deuteration level of the hCC fragments in the presence (black color) and in the absence (blue color) of Cyst10 antibody.*

Figure S3 *Deuteration level of the hCC fragments in the presence (black color) and in the absence (blue color) of Cyst28 antibody*

Figure S4 *Deuteration level of the hCC fragments in the presence (black color) and in the absence (blue color) of polyclonal NAbs antibody.*
